# Effects of acute bouts of physical activity on children's attention: a systematic review of the literature

**DOI:** 10.1186/2193-1801-3-410

**Published:** 2014-08-05

**Authors:** Mirka Janssen, Huub M Toussaint, Willem van Mechelen, Evert ALM Verhagen

**Affiliations:** Academy for Physical Education, Technical University of Applied Sciences of Amsterdam, Amsterdam, the Netherlands; Department of Public and Occupational Health, EMGO+ Institute for Health and Care Research, VU University Medical Centre, Amsterdam, the Netherlands

**Keywords:** Exercise, Sport, Cognitive performance, Cognitive control, Concentration, Systematic review, Physical activity

## Abstract

The aim of this review was to describe the effects of acute bouts of physical activity on attention levels of children. A systematic review was performed of English studies from searches in PubMed, Sportdiscus and PsycINFO from 1990 to (May) 2014 according to the PRISMA statement. Only prospective studies of children aged 4-18 years old were included, detailing acute effects of physical activity bouts with the primary outcome attention.

One reviewer extracted data on the study characteristics. Two reviewers conducted the methodological quality assessment independently using a criteria checklist, which was based on the Downs and Black checklist for non-randomised studies.

Overall the evidence is thin and inconclusive. The methodological differences in study sample (size and age), study design and measurement of attention make it difficult to compare results.

There is weak evidence for the effect of acute bouts of physical activity on attention. More experimental studies with a comparable methodology, especially in the school setting, are needed to strengthen this evidence.

**Electronic supplementary material:**

The online version of this article (doi:10.1186/2193-1801-3-410) contains supplementary material, which is available to authorized users.

## Introduction

Schools have been recognised as key settings for promoting physical activity (PA) in children, because children spend a large part of their regular days in school (Biddle et al. 1998). Therefore, schools are frequently requested to implement different physical activity programs. However, schools have the primary priority to improve cognition and are under pressure to improve academic scores. This often results in additional time for cognitive learning and less time for physical education classes or recess (Center for Education Policy, 2007). Nevertheless, Ahamed et al. ( 2007) concluded that decreasing time spent in PA does not improve academic performance. Furthermore, a recent review concluded strong evidence for a significant positive relationship between PA and academic performance (Singh et al. 2012).

However, the evidence from this review is based on cross-sectional studies and does not give insight in the complex relationship between PA and academic performance. Fortunately, the literature-base on the acute effect of PA on the underlying cognitive processes of academic performance is growing. Hillman et al. ( 2011) found in their review a positive effect of acute PA on brain health and cognition in children, but concluded it was complicated to compare the different studies due to the different outcome measures (e.g. memory, response time and accuracy, attention, and comprehension). Therefore, this review focuses on the sole outcome measure ‘attention’ as a mediator for cognition and achievement.

Attention is defined as the ability to resist distraction. Attention acts as a ‘gate’ into working memory, regulating the flow of sensory information into conscious awareness (Baddeley, 2001). Attention is important for several aspects of learning and memory storage; attention is required when learning something (to encode the information) but also when recalling a memory (Hillman et al. 2003). Deficits in attention are associated with poorer academic performance (Aronen et al. 2005).

To our knowledge, no systematic review on the acute effect of PA focusing on attention has been published. In this systematic review, experimental and observational studies examining the effect of acute bouts of PA on attention in children were included.

## Methods

### Review protocol

The PRISMA-statement for reporting systematic reviews and meta-analyses of studies that evaluate health care interventions (Liberati et al. 2009) was used as a guideline to conduct this review. Prior to the review, a review protocol was made in which pre-specified outcomes of primary interest, the methodology of data extraction on these outcomes and the methodological quality assessment was described (Additional file 1).

#### Eligibility criteria

**Population** Only prospective studies (experiments and observations) that were conducted with children were included in the review. In the protocol children were defined as the age group between 4 and 18 years old. Since the growth factor BDNF is also associated with metabolism (Pedersen et al. 2009) and behavioural disorders (e.g. ADHD) with inattentiveness, only studies with healthy participant groups were included. Studies with specific groups (e.g. children with obesity or diabetes 2, children with ADHD or depression) were excluded, as these characteristics may be confounders.

**PA bout** Studies with a short PA bout (i.e. max 45 minutes) and various levels of PA intensity were included. PA bouts could be performed during a physical education lesson, in-between lessons, at the playground, or as an energizer during class. The PA bouts could be performed with or without equipment or apparatus.

**Outcomes** Only studies with an outcome measure of some sort of attention (e.g. attention, on-task behaviour, neuroelectric attentional performance) were included. Studies that focused on other cognitive tasks, (e.g. short-term or long-term memory, successive processing etc.) were excluded.

**Information sources** Studies were identified by searching electronic databases (PubMed, Sportdiscus and PsycINFO) from 1990 to May 2014. The search consisted of three elements, which were combined in the final search strategy: (1) physical activity (i.e. physical activity, leisure activity, exercise, physical fitness, sport, cycling, walking and training) (2) attention (i.e. attention, on task performance, attentional performance, cognitive control, executive control, concentration) and (3) age (i.e. infant, child and adolescent). Medical Subject Headings (MeSH) were available for physical activity (all synonyms), attention and age (all synonyms). MeSH terms and free text words were used in all databases (Additional file 2). In addition, a hand search was done in reference lists of identified studies for relevant literature.

**Study selection** All experimental and observational studies, which were full-text articles published after 1990 in English peer-reviewed journals were included. One reviewer (MJ) screened all titles and abstracts and in case of uncertainty, the full article was screened.

**Data collection process** One reviewer (MJ) extracted data on the study population, the study design, the PA bout, measure of attention, and on the main results. Two reviewers (MJ and EV) independently conducted the methodological quality assessment and disagreements were resolved by discussion. For this assessment a criteria checklist (based on the Downs and Black checklist for non-randomised studies (Downs and Black, 1998) was used. This checklist consists of 27 items and contains items to assess the quality of the reporting, the external and internal validity of the study and the study power. The criteria answer format included yes (1) and no or unable to determine (both coded 0). A criterion was scored as ‘not applicable’ (NA), when the criterion was not relevant for the study design.

One criterion needed clarification of interpretation before scoring the studies. The criteria ‘Was compliance with the interventions reliable’ was scored with ‘0’ when no attempt was made to define the type, duration and level of intensity (for example with heart rate monitors) of PA.

In order to establish the validity and proper use of this set of predefined criteria, the inter-rater agreement, expressed as Cohen’s κ, was calculated.

## Results

### Study selection

**Figure 1:**
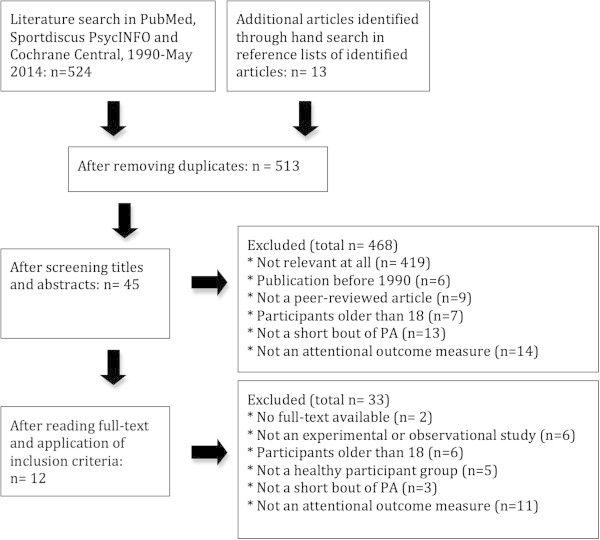
**Flow diagram of the selection procedure.**

The systematic literature search combined by hand searches revealed 537 studies. After excluding duplicates (n = 24), titles and abstracts of 513 studies were screened for eligibility, of which 12 were included in this review. Figure 1 shows the flow diagram of the selection process, with reasons for exclusions at each stage.

### Methodological assessment

In total 12 studies were screened on 25 criteria (Additional file 3). Table 1 provides the methodological assessment for each individual study. The reviewers scored different on 33 of 250 items, equalling to a Cohen’s κ of 0.74. This is considered reasonable to good (Lantz and Nebenzahl, 1996).

**Table 1:** **Results of the methodological quality assessment**

Criteria	#1 Raviv & Low, 1990	#2 Caterino & Polak, 1999	#3 Mahar et al., 2006	#4 Budde et al., 2008	#5 Cereatti et al., 2009	#6 Hillman et al., 2009	#7 Grieco et al., 2009	#8 Stroth et al., 2009	#9 Drollette et al., 2012	#10 Pirrie & Lodewyk, 2012	#11 Pontifex et al., 2013	#12 Drollette et al., 2014
**Objective**	1	1	1	1	1	1	1	1	1	1	1	1
**Main outcomes**	1	1	1	1	1	1	1	1	1	1	1	1
**Participant characteristics**	0	0	0	1	0	1	1	1	1	0	1	1
**Interventions of interest**	0	0	0	0	1	1	0	1	1	1	1	1
**Confounders described**	0	0	0	1	1	1	0	1	1	0	1	1
**Main findings**	1	1	1	1	1	1	1	0	1	1	1	1
**Random variability**	1	1	1	1	1	1	1	1	1	1	1	1
**Characteristics of lost to follow-up participants**	0	0	0	0	1	1	0	1	1	0	1	1
**Actual probability values given**	1	1	0	1	1	1	0	0	1	1	1	1
**Representative population**	0	0	0	0	0	0	0	0	0	0	0	0
**Representative participants**	0	0	0	0	0	0	0	0	0	0	0	0
**Blinding of participants**	0	0	0	0	0	0	0	0	0	0	0	0
**Blinding of test leaders**	0	0	0	0	0	0	1	0	0	0	0	0
**Control and intervention condition described (type, duration and level PA)**	0	0	0	1	0	1	0	1	1	0	1	1
**Length of follow-up same for intervention and control group**	0	NA	0	1	NA	0	1	1	0	1	0	0
**Statistical tests appropriate**	1	1	1	1	1	1	1	1	1	1	1	1
**Compliance to intervention measured**	0	0	0	1	1	1	0	1	1	1	1	1
**Main outcome measures accurate**	1	1	1	1	1	1	1	0	1	1	1	1
**Intervention and control group recruited from the same population**	1	1	1	1	0	0	1	0	1	1	0	1
**Intervention and control group recruited over the same period of time**	1	1	1	1	0	0	1	1	1	1	1	1
**Randomisation**	0	1	1	1	0	0	1	1	1	0	1	1
**Randomisation assignment concealed**	0	0	0	0	0	0	0	0	0	0	0	0
**Adjustment for confounding**	0	0	0	0	0	0	0	1	0	0	0	0
**Losses to follow-up taken into account**	0	0	0	0	1	1	0	1	0	1	0	0
**Power analysis provided**	0	0	0	0	0	0	0	0	0	0	0	0
	#1	#2	#3	#4	#5	#6	#7	#8	#9	#10	#9	#12
**Total score**	9	10	9	15	11	14	12	15	16	13	15	16
**Percentage (%)**	**36**	**40**	**36**	**60**	**44**	**56**	**48**	**60**	**64**	**52**	**60**	**64**

In 22 of the items, there was initial disagreement as to whether an item was described in the studies. In the other 11 items, reviewers disagreed; mainly regarding the representativeness of the study sample, participant characteristics and possible confounders (e.g. ADHD, BMI, physical fitness). After discussing the differences, agreement was reached for all differences. A number of the disagreement issues are described below.

A ‘0’ was given when no participant characteristics were given, but also when participant characteristics were partly described. For example in one study (Cereatti et al. 2009) different important characteristics were given, but no information was available on BMI, level of intensity of PA or type of sport. Type of sport could be a modifier for socio-economic status. In one study (Budde et al. 2008) the intervention (coordinative exercises) was clearly described, but not the control condition. The control condition was described as ‘teachers instructed the students to exercise at a moderate intensity without any specific coordinative request’ and it remains unclear which exercises were performed that were not of coordinative character.

A note has to be made on the criterion ‘Was an attempt made to blind study objects to the intervention they have received’. This criterion was scored ‘0’ in every study and it must be noted that blinding participants from a PA intervention is practically impossible.

### Synthesis of results based on the methodological assessment

Overall, the studies of Drollette et al. ( 2012), and Drollette et al. ( 2014) had the highest methodological score. From the studies in a school setting Budde et al. ( 2008) had the highest score. The study of Drollette et al. ( 2012) reported maintenance of attention level after exercise compared to seated rest and the study of Drollette et al. ( 2014) reported an improvement in attention level. Budde et al. ( 2008) reported a positive effect on attention through coordination exercises at a moderate PA level as compared to a normal PE lesson of the same intensity level.

### Study characteristics

Table 2 provides a summary of the studies included in the review with regard to the main characteristics. Ten experimental studies (Raviv and Low, 1990; Caterino and Polak, 1999; Budde et al. 2008; Cereatti et al. 2009; Hillman et al. 2009; Stroth et al. 2009; Drollette et al. 2012; Pirrie and Lodewyk, 2012; Pontifex et al. 2013; Drollette et al. 2014) and two observational studies, Mahar et al. ( 2006); Grieco et al. 2009) were included, with in total 916 participants in the age range of 7 to 17 years old.

**Table 2:** **Main characteristics of the included studies USA = United States of America; n = number of participants; yrs = years old; PA = physical activity; PE = physical education; vs = versus; min = minutes; HR = heart rate; HRR = Heart Rate Reserve; MVPA = moderate to vigorous intensity PA; RT = reaction time; ADHD = attention-deficit/hyperactivity disorder**

#	Study	Country	Population	Design; setting	PA assessment	PA type; duration; level	Attention measure	Main results
			(n; age)					
1	Raviv & Low, 1990	Israel	n = 69; n boys and girls unknown;	Experiment; school-setting	None	PE class vs science class; unknown; unknown	D2 (visual selective attention, information processing speed, ability to concentrate)	Higher scores at the end of lesson, no significant difference between classes (p = 0.47)
			11–12 yrs					
2	Caterino & Polak, 1999	USA	n = 177; n boys and girls unknown;	Experiment; school-setting	None	Stretching and aerobic walking vs classroom task; 15 min.; unknown	Woodcock-Johnson Test of Concentration	Significant difference only for 9–10 years old children (p = 0.05)
			7–10 yrs					
3	Mahar et al., 2006	USA	n = 243; n boys and girls unknown;	Observation of 12 weeks	Number of steps (pedometer)	Energizers; 10 min; unknown	Observation of on-task behaviour	Significant improvement (8%, p = 0.017), low performers 20%
			8–11 yrs	Daily intervention; school-setting				
4	Budde et al., 2008	Germany	n = 115; 80 boys, 19 girls;	Experiment; school-setting	HR	Normal PE class vs coordinative exercises; 10 min; moderate	D2 (visual selective attention, information processing speed, ability to concentrate)	Significant improvement after coordinative exercises (p < 0.01)
			13–16 yrs					
5	Cereatti et al., 2009	Italy	n = 24; 24 boys, 0 girls;	Experiment; laboratory	HR	Bicycle ergometer; duration unknown (as long as attention measure lasted); 60%HRR	Computerized visual attention task	Significant improvement in RT (p < 0.023)
			14–17 yrs					
6	Hillman et al., 2009	USA	n = 20; n boys and girls unknown;	Experiment; laboratory	HR	Treadmill; 20 min; 60% HR max	A modified flanker task (inhibitory control), combined with EEG	Effect on cognitive control of attention; Significant improvement of accuracy (p = 0.008), no improvement in RT
			9–10 yrs					
7	Grieco et al., 2009	USA	n = 97; n boys and girls unknown;	Observation of 1 school year. Intervention 4 days a week; school-setting	Observation of PA level;	PA during classroom task; 10–15 min; MVPA	Time on task (TOT)	No decrease of TOT after PA (significant difference with inactive lesson, p < 0.001)
			7–8 yrs		Number of steps (pedometer)			
8	Stroth et al., 2009	Germany	n = 33; 20 boys and 13 girls;	Experiment; laboratory	HR	Bicycle ergometer; 20 min; 60% HRmax	A modified flanker task (task preparation and response inhibition), combined with EEG	Acute moderate PA was not related to executive control (attention among others; p > 0.76)
			13–14 yrs					
9	Drollette et al., 2012	USA	n = 36; 16 boys and 20 girls;	Experiment; laboratory	HR	Treadmill; 20 min; 60% HRmax	A modified flanker task (inhibitory control)	Effect on attention after walking, not during walking. Maintenance of accuracy (p = 0.01) after PA vs. seated rest, not in RT
			9–11 yrs					
10	Pirrie & Lodewyk, 2012	Canada	n = 40; 22 boys; 18 girls	Experiment; school-setting	HR (in half of the children)	45 min PE lesson; 28–30 min in MVPA (≥65% HRmax)	Cognitive Assessment System (planning, attention, simultaneous processing, successive processing)	No significant effect on attention
			9–10 yrs					
11	Pontifex et al., 2013	USA	n = 20 (other 20 non-eligible: children with ADHD ); 14 boys, 6 girls; 8–10 yrs	Experiment; laboratory	HR	Treadmill; 20 min; 65-75% HRmax	A modified flanker task (inhibitory control), combined with EEG	Effect on cognitive control of attention; Significant improvement of accuracy (p = 0.011), no improvement in RT. Better improvement in children with ADHD
12	Drollette et al., 2014	USA	n = 40; 13 boys and 27 girls;	Experiment; laboratory	HR	Treadmill; 20 min; 60-70% HRmax	A modified flanker task (inhibitory control), combined with EEG	Effect on cognitive control of attention; Significant improvement of accuracy (p = 0.003), no improvement in RT. Better improvement in low performers
			8–10 yrs					

Six studies were performed in a laboratory (Cereatti et al. 2009; Hillman et al. 2009; Stroth et al. 2009; Drollette et al. 2012; Pontifex et al. 2013; Drollette et al. 2014) and six in a school setting. Of these six studies, three studies (Raviv and Low, 1990; Caterino and Polak, 1999; Pirrie and Lodewyk, 2012) examined the difference between a classroom task and an active lesson. One study (Budde et al. 2008) examined the difference between two active lessons with different activity types, one study (Mahar et al. 2006) examined the effect of energizers (i.e. short bouts of PA in the classroom) and one study (Grieco et al. 2009) examined the effect of exercise during a cognitive task.

The PA bouts differed amongst studies in type, duration and level. In seven studies an aerobic type of PA was performed (Caterino and Polak, 1999; Cereatti et al. 2009; Hillman et al. 2009, Stroth et al. 2009 and Drollette et al. 2012; Pontifex et al. 2013; Drollette et al. 2014), in one study coordinative exercises were compared to a normal PE lesson (PA type of exercises unknown) (Budde et al. 2008) and in four studies the PA type was not specified (Raviv and Low, 1990; Mahar et al. 2006; Grieco et al. 2009; Pirrie and Lodewyk, 2012). The duration of the PA bout varied from 10 to 45 minutes. Assessment of PA level was done in all six laboratory studies (Cereatti et al. 2009; Hillman et al. 2009; Stroth et al. 2009; Drollette et al. 2012; Pontifex et al. 2013; Drollette et al. 2014) and in two studies performed in the school setting (Budde et al. 2008; Pirrie and Lodewyk, 2012). The PA level varied around 60% of the maximum heart rate in all six studies, which corresponds to a moderate intensity level of PA. This was either theoretically estimated by 220-age (Budde et al. 2008; Cereatti et al. 2009) or measured by a direct VO2max test (Hillman et al. 2009; Stroth et al. 2009; Drollette et al. 2012; Pirrie and Lodewyk, 2012; Pontifex et al. 2013; Drollette et al. 2014). Attention was measured by different measures. In the studies performed in a school setting, four different measurements were done (D2-test, observation of on-task behaviour or time on task, the Woodcock-Johnson test of Concentration and the Cognitive Assessment System (CAS)). In the laboratory studies, the measurement of attention was more comparable; either a computerized visual attention task or (modified) flanker tasks were used.

The main results were inconclusive. Five of the six laboratory studies found a significant effect on cognitive control of attention. One laboratory study (Cereatti et al. 2009) found a significant improvement in reaction time, one study showed a maintenance of accuracy of task (Drollette et al. 2012) and three studies showed a significant improvement on accuracy on the task and not on reaction time (Hillman et al. 2009; Pontifex et al. 2013; Drollette et al. 2014).

Regarding the studies in a school setting, there was no overall significant difference in attention after an active lesson and a classroom lesson. However, analyses of subgroups showed significant results. Caterino and Polak ( 1999) found a significant effect of PA only for 9–10 years old children and Budde et al. ( 2008) found a significant effect only after coordinative exercises (for example bouncing a ball, while balancing). Performing energizers led to a significant improvement of time on task (i.e. the time in verbal or motor behaviour that followed the class rules and was appropriate to the learning situation) after performing the energizers (Mahar et al. 2006), but when these energizers were performed during a classroom task, no improvement was found (Grieco et al. 2009).

It must be noted that no study provided a power analysis and therefore the lack of significant results can be caused by insufficient power to reject the null hypothesis.

## Discussion

In this systematic review, 12 experimental and observational studies were included that examined the effect of acute bouts of PA on attention in children. Due to methodological differences in study sample (size and age), study design and measurement of attention it was difficult to compare results. These differences are discussed below.

### Population

Sample characteristics between studies differed and made it difficult to compare the results. For example the age range between studies ranged from 7 to 17 years. Caterino and Polak ( 1999) found significant effect of PA only for 9–10 year old children, no effects were found for 7–8 years old children, indicating the differences in outcomes between ages and the difficulty in comparing the various included studies. Children undergo rapid, process-specific changes in cognitive development. Thus, age may influence potential mechanisms for the effects of PA on attention. There was no evidence of influence of gender on the acute effect of PA on attention. However, most studies did not examine gender differences.

### PA bouts

In most studies an aerobic type of PA was included, of which 4 (from 7) found a positive effect on attention. Budde et al. ( 2008) compared a PA bout consisting of coordinative exercises to a normal PE lesson of the same intensity level and found a significant difference between the two PA bouts in favour of the coordinative exercise condition. Coordinative exercises might lead to pre-activation of parts of the brain, which are also responsible for mediating functions like attention. This explanation is further supported by a study on cognitive flexibility, which demonstrated that cortical transcranial magnetic stimulation manipulates subcortical cognitive functions (van Schouwenburg et al. 2012). Thus, type of activity may have influence on the acute effect of a PA bout on attention.

The length of PA bouts differed among studies, varying between 10 to 45 minutes. In the majority of studies that included a short PA bout of maximum of 20 minutes, a significant effect of PA on attention was found. In contrast, in the study from Pirrie and Lodewyk ( 2012) no effect was found after a 45 minutes PA bout. This indicates that duration of PA may influence the effect of PA on attention.

Overall all studies included a short bout of moderate intensity PA. In the study of Pirrie and Lodewyk ( 2012), 67% of the PA bout was strenuous (>65% HRmax). This study showed no effect of PA on attention. Arguably, intensity of PA influences the effect of PA on attention and the effect of PA on attention may follow and inverted U relationship. The inverted-U-hypothesis (Yerkes and Dodson, 1908) states that cognitive performance is optimally enhanced with a moderate level of arousal (McMorris and Graydon 2000) and that PA can increase arousal level. The relation between the intensity of PA and arousal in mice follows an inverted U, with an optimum at moderate PA intensity (Rhodes et al. 2003). Also for human adults, the optimal level of arousal seems to be at moderate intensity PA (Brisswalter et al. 2002). Arguably, this optimal level is the same in children because despite the fact that children undergo rapid, process-specific changes in cognitive development, attentional control is fully developed by the age of 7 (Rueda et al. 2005).

There are several suggested mechanisms for a positive association between PA and cognitive skills, which are mainly explained by neuropsychological improvements (e.g. increased blood flow to the brain (Jorgensen et al. 2000), increased levels of hormones which results in a reduction of stress (Fleshner, 2000), and increased growth factors for creating new cells (van Praag et al. 1999). Due to these growth factors, attention, stimulus selection, and decision making are improved (Griffin et al. 2011). BDNF is one of these growth factors and is mainly found in the pre-frontal cortex, basal forebrain and hippocampus (Griffin et al. 2011) where decision making takes place (i.e. priority is given to important information and distraction is eliminated).

### Outcome measures

Although this systematic review focused on one outcome measure i.e. attention, it remains difficult to compare the outcome of the studies. The definition of attention is not unambiguous. Theoretically a distinction is made between selective attention (the ability to complete a task without being distracted by other stimuli that are being presented), divided attention (the ability to complete multiple tasks at once) and sustained attention (the ability to stay focused on a task for a long time) (de Jong, 1991). In addition, attention is always involved in other cognitive processes, which makes the measurement of attention difficult (de Jong, 1991). Therefore, a variety of attention tests are available, which also reflects the differences in methodology of the studies in this review.

In the studies performed in a school setting, four different measurements of attention at a behavioural level were employed; the D2-test, which measures visual selective attention, information processing speed and the ability to concentrate; observation of on-task behaviour or time on task, which is a measure of sustained attention; the Woodcock-Johnson Test of Concentration, which measures selective attention; and the Cognitive Assessment System (CAS), in which the attention test was a Stroop-like task, i.e. a measure of selective attention and inhibitory control.

In the laboratory studies, the measurement of attention was done at the neural level; either a computerized visual attention task, which measures reaction time and accuracy; or a (modified) flanker task, which measures response speed, accuracy and changes in the speed and accuracy of information processing, was used to measure attention. In these tests, inhibitory control is an important factor.

In four laboratory studies, the modified flanker task was combined with EEG. An EEG shows neuropsychological changes, which are reflected in the amplitude and the latency of the P3 (an event related potential (ERP) component elicited in the process of decision making). A higher amplitude reflects a greater resource accuracy by a greater attentional allocation (Polich, 1987) and an increased latency reflects longer processing time (Duncan-Johnson, 1981).

The results from the studies using a flanker task showed a positive effect from a short PA bout on attention, except for the study from Stroth et al. ( 2009). However, Hillman et al. ( 2011) stated that their EEG measurement was not performed at the most appropriate region of the scalp.

#### Summary of evidence

Overall the evidence is weak and inconclusive due to methodological differences. Although we focused on one outcome measure, the methodological differences in study sample (size and age), study design, and measurement of attention make it difficult to compare results.

Although the laboratory studies are more comparable, there is limited generalizability of the results to the school setting. The few studies that have been conducted within a school setting are less comparable, due to differences in methodology. Although laboratory based research can allow for greater scientific rigour than field based research, more methodologically comparable studies in the school setting are needed to strengthen this evidence. Therefore, it is necessary to create a robust knowledge base about the duration and intensity of the acute bout of PA that influences the effect on attention and also about the measurement of attention in a school setting.

#### Limitations

This study concerns the results of a systematic review on studies which evaluated the effect of a single acute bout of PA on attention. A limitation is of this review is that no meta-analysis could be performed. This might have been possible for the laboratory studies, in which the measurements were comparable.

Furthermore, the measurement of the quality of the studies depends on the interpretation of the reviewers and the choice for the checklist. A different checklist could have given different results.

Although we screened reviews and reference lists, the possibility exists of publication bias, which leads to an overestimation of a potential positive effect from physical activity on attention. On the other hand, a few studies with no effect on attention were selected for this review.

The synthetic approach could give a false impression of homogeneity, in particular with regard to measurement of intensity of PA and attention. Provision of the details in the tables will give insight into heterogeneity.

## Electronic supplementary material

Additional file 1: **Review protocol.** (DOCX 14 KB)

Additional file 2: **Full search strategy.** (DOCX 14 KB)

Additional file 3: **Criteria checklist for the methodological assessment.** (DOCX 20 KB)
