# Supplementary material for: Effects of acute bouts of physical activity on children's attention: a systematic review of the literature
Source: Springerplus. 2014 Aug 5;3:410. doi: 10.1186/2193-1801-3-410 (PMC4132441; doi:10.1186/2193-1801-3-410)
Supplement: Supplementary file 1 — Additional file 1: Review protocol. (DOCX 14 KB) [file 40064_2014_1114_MOESM1_ESM.docx]

Additional file 1 Review protocol

**Outcomes of primary interest**

| Attention | Selective attention |
| --- | --- |
|  | On-task behaviour |
|  | Neuro-electric attentional performance |

| **Inclusion criteria** | **Exclusion criteria** |
| --- | --- |
| Outcome: attention | When an inclusion criteria is not met |
| Age 4-18 years old | Participant group with obesity |
| Short PA bout (<30 min) | Participant group with Diabetes 2 |
| PA setting: in between lessons, at the playground, energizer during class, in a laboratory | Participant group with ADHD |
| PA equipment: with or without | Participant group with depression |
| Full-text |  |
| English, peer-reviewed journal |  |
| 1990-may 2014 |  |

**Methodology of data extraction on these outcomes**

| **#** | First Author, year of publication |
| --- | --- |
| **Study Population** | Number of participants, number of boys and girls, age |
| **Country** | Country |
| **Design** | Experiment or observation |
| **PA measure** | Heart rate monitor or pedometer or accelerometer |
| **PA type; duration; level** | Kind of exercise; total minutes of PA, intensity level of PA |
| **Attention measure** | Measurement method of outcome (attention) |
| **Main results** | Difference / improvement / effectiveness |

**Methodological quality assessment**

The methodological quality assessment was based on the Downs and Black checklist for non-randomised studies [Downs & Black, 1998]. The checklist can be accessed at: <http://bjsm.bmj.com/content/suppl/2011/12/22/bjsports-2011-090428.DC1/bjsports-2011-090428_ds2.pdf>

This checklist consists of 27 items and contains items to assess the quality of the reporting, the external and internal validity and the power.

Two criteria (‘Have all adverse events that may be a consequence of the intervention been reported’ and ‘If any of the results of the study were based on data dredging, was this made clear’) will be left out, because these are not relevant for the selected studies.

One criteria needed clarification of interpretation before scoring the studies. The criteria ‘Was compliance with the interventions reliable’ will be scored with ‘0’ when no attempt was made to define the level of PA (for example with a heart rate monitor) and with ‘1’ when this was done.

The methodological quality assessment will be conducted independently by two reviewers (MJ and EV), and disagreements resolved by discussion.
