# Supplementary material for: Effects of acute bouts of physical activity on children's attention: a systematic review of the literature
Source: Springerplus. 2014 Aug 5;3:410. doi: 10.1186/2193-1801-3-410 (PMC4132441; doi:10.1186/2193-1801-3-410)
Supplement: Supplementary file 2 — Additional file 2: Full search strategy. (DOCX 14 KB) [file 40064_2014_1114_MOESM2_ESM.docx]

**Additional file 2 Full search strategy**

The search consisted of three elements, which were combined in the final search strategy.

(1) Physical activity (i.e. physical activity, leisure activity, exercise, physical fitness, sport, cycling, walking and training)

(2) Attention (i.e. attention, on task performance, attentional performance, cognitive control, executive control, concentration)

(3) Age (i.e. infant, child and adolescent).

**Search Strategy**

| **Example in Pubmed** | [**http://www.ncbi.nlm.nih.gov/pubmed**](http://www.ncbi.nlm.nih.gov/pubmed) |
| --- | --- |
| (physical activity OR fitness OR physical fitness OR exercise OR sports OR cycling OR walking OR training OR physical education OR recess) AND (attention OR on task performance OR attentional performance OR cognitive control OR executive control or concentration) AND (infant OR infant OR child* OR adolescent) AND (randomized controlled trials OR observation* OR comparative study OR exp evaluation studies OR follow-up studies OR prospective studies) | |
| Limits Activated: Humans, English | |
