# Supplementary material for: Effects of acute bouts of physical activity on children's attention: a systematic review of the literature
Source: Springerplus. 2014 Aug 5;3:410. doi: 10.1186/2193-1801-3-410 (PMC4132441; doi:10.1186/2193-1801-3-410)
Supplement: Supplementary file 3 — Additional file 3: Criteria checklist for the methodological assessment. (DOCX 20 KB) [file 40064_2014_1114_MOESM3_ESM.docx]

**Additional file 3 Criteria checklist (based on the Downs and Black checklist for non-randomised studies)**

| **Item** | Description | Note (agreement / interpretation reviewers) | Score |
| --- | --- | --- | --- |
| **Reporting** | | | |
| **1** | Is the hypothesis/aim/objective of the study clearly described? |  | Y=1/N=0 |
| **2** | Are the main outcomes to be measured clearly described in the Introduction or Methods section? |  | Y=1/N=0 |
| **3** | Are the characteristics of the patients included in the study clearly described? | Inclusion and / or exclusion criteria. More than age and gender. A note on at least two of the following characteristics must be made: body mass index, neurological diseases, attentional disorders, physical disabilities, physical activity or SES = Y. | Y=1/N=0 |
| **4** | Are the interventions of interest clearly described? | Detailed description of the intervention AND the control condition. | Y=1/N=0 |
| **5** | Are the distributions of principal confounders in each group of subjects to be compared clearly described? | A list of confounders is described. | Y=2/Partial=1/N=0 |
| **6** | Are the main findings of the study clearly described? |  | Y=1/N=0 |
| **7** | Does the study provide estimates of the random variability in the data for the main outcomes? | Reporting of standard error, standard deviation or confidence intervals | Y=1/N=0 |
| **8** | Have the characteristics of patients lost to follow-up been described? | No losses or small losses = Y. No reporting = N. | Y=1/N=0 |
| **9** | Have actual probability values been reported (eg 0,035 rather than <0,05) for the main outcomes except where the probability value is less than 0,001 |  | Y=1/N=0 |

| **Item** | Description | Note | Score |
| --- | --- | --- | --- |
| **External validity** | | | |
| **10** | Were the subjects asked to participate in the study representative of the entire population from which they were recruited? | The source population must be indentified and the selection of the participants must be described. | Y=1/N=0/UnabletoDetermine=0 |
| **11** | Were those subjects who were prepared to participate representative of the entire population from which they were recruited? | The proportion of those asked who agreed to participate must be described. | Y=1/N=0/UnabletoDetermine=0 |
| **12** | Were the staff, places and facilities where the patients were treated, representative of the treatment the majority of the patients received? | Study performed in a school setting = Y. Study performed not in a school setting (e.g. laboratory) = N. | Y=1/N=0/UnabletoDetermine=0 |
| **Internal validity (BIAS)** | | | |
| **13** | Was an attempt made to blind study subjects to the intervention they have received |  | Y=1/N=0/UnabletoDetermine=0 |
| **14** | Was an attempt made to blind those measuring the main outcome of the intervention |  | Y=1/N=0/UnabletoDetermine=0 |
| **15** | In trials and cohort studies, do the analyses adjust for different lengths of follow-up of patients, or in case-control studies, is the time period between the intervention and outcome the same for cases and controls? | Same follow-up or adjusted for different lengths of follow-up = Y. Differences in follow-up = N. | Y=1/N=0/UnabletoDetermine=0 |
| **16** | Were the statistical tests used to assess the main outcomes appropriate? |  | Y=1/N=0/UnabletoDetermine=0 |
| **17** | Was compliance with the interventions reliable | When an attempt was made to measure the compliance with a heart-rate monitor = Y. When no heart rate monitor was used = N. | Y=1/N=0/UnabletoDetermine=0 |
| **18** | Were the main outcome measures used accurate (valid and reliable) |  | Y=1/N=0/UnabletoDetermine=0 |

| **Item** | Description | Note | Score |
| --- | --- | --- | --- |
| **Internal validity (confounding)** | | | |
| **19** | Were the patients in different intervention groups (trials and cohort studies) or were the cases and controls (case-control studies) recruited from the same population | Recruited from the same school = Y. | Y=1/N=0/UnabletoDetermine=0 |
| **20** | Were study subjects in different intervention groups (trials and cohort studies) or were the cases and controls (case-control studies) recruited over the same period of time? |  | Y=1/N=0/UnabletoDetermine=0 |
| **21** | Were study subjects randomised to intervention groups? |  | Y=1/N=0/UnabletoDetermine=0 |
| **22** | Was the randomised intervention assignment concealed from both patients and health care staff until recruitment was complete and irrevocable? | Non-randomised studies = N. | Y=1/N=0/UnabletoDetermine=0 |
| **23** | Was there adequate adjustment for confounding in the analyses from which the main findings were drawn? |  | Y=1/N=0/UnabletoDetermine=0 |
| **24** | Were losses of patients to follow-up taken into account? | Small loses = Y. Not reported = UD = 0. | Y=1/N=0/UnabletoDetermine=0 |
| **Power** | | | |
| **25** | Did the study have sufficient power to detect a clinically important effect where the probability value for a difference being due to chance is less than 5%? | Sample sizes calculated = Y. | Y=1/N=0 |
